# Impact of nutrient supplementation on maternal nutrition and child growth and development in Sub‐Saharan Africa: the case of small‐quantity lipid‐based nutrient supplements

**DOI:** 10.1111/mcn.12960

**Published:** 2020-12-21

**Authors:** Seth Adu‐Afarwuah

**Affiliations:** ^1^ Department of Nutrition and Food Science University of Ghana Accra Ghana

**Keywords:** Sub‐Saharan Africa, micronutrient deficiencies, micronutrient supplementation, home‐fortification, small‐quantity lipid‐based nutrient supplements

## Abstract

Micronutrient deficiencies remain common among women and children in Sub‐Saharan Africa (SSA); in pregnant/lactating women, the intakes of essential fatty acids may also be low. Enriching home‐prepared foods with small‐quantity lipid‐based nutrient supplements (SQ‐LNSs) is a promising new strategy of delivering additional micronutrients, essential fatty acids and good quality protein to women and children. This narrative review aimed to examine the impact of SQ‐LNSs supplementation among women and infants and young children in SSA, and to discuss the differential impact of SQ‐LNS consumption across different settings.

Papers reporting randomized trials conducted in SSA in which apparently healthy women and/or ≥6‐mo‐old children received SQ‐LNSs were identified through electronic and manual searches.

Prenatal SQ‐LNS consumption reduced the prevalence of low gestational weight gain in Ghana when compared with multiple micronutrients supplementation, and was associated with poorer iron/hemoglobin status when compared with iron‐plus‐folic acid supplementation. SQ‐LNSs received alone or as intervention package improved infant/child growth in two trials in Ghana and one trial each in Burkina Faso, Kenya, Zimbabwe and South Africa, but had no impact on growth in two trials in Malawi. SQ‐LNSs supplementation improved motor development in Ghana, Burkina Faso, Malawi, Kenya, and South Africa, but had no impact on language, socio‐emotional, and executive functions in Ghana and Malawi and on Griffiths’ developmental scores in Malawi.

SQ‐LNSs may contribute to improving child growth in SSA. More research is needed to determine the iron level in SQ‐LNSs effective for improving both maternal hemoglobin/iron status and birth outcomes.

**Key messages**

- Small‐quantity lipid‐based nutrient supplements include the family of multiple micronutrient‐fortified semi‐solid pastes typically given at a dose of about 20 g/d or 110 kcal/day for the purpose of preventing undernutrition.
- Prenatal SQ‐LNS consumption reduced the prevalence of low gestational weight gain (GWG) in Ghana. When given alone or as part of a package of interventions, SQ‐LNSs consumption has been associated with improved infant or child growth in several settings in Sub‐Saharan Africa.
- The impact of SQ‐LNS supplementation in promoting fetal and child growth in SSA may be context‐specific. Such interventions might have a greater impact if they address the complex array of underlying factors that contribute to growth faltering.

**Abbreviations**
GWG: Gestational weight gain
IFA: Iron and folic acid
LNS: Lipid‐based Nutrient Supplement
MMN: Multiple Micronutrients
SQ‐LNS: Small‐quantity lipid‐based nutrient supplements
SSA: Sub‐Saharan Africa

## 1 INTRODUCTION

Across Sub‐Saharan Africa (SSA), the prevalence of undernutrition (eg anemia, micronutrient deficiencies, and stunting) among women and children has been consistently high for decades. According to 2017 estimates, about 47% of pregnant women in the region are anemic and about 14% have vitamin A deficiency, which represent only relatively small reductions in prevalence compared with estimates for the early 1990s (Ritchie & Roser, 2019). Likewise, between the early 1990s and 2017, the prevalence of anemia in children under 5 years of age decreased from about 76% to only 60%, and stunting rates from 49% to 34% (Ritchie & Roser, 2019; Roser & Ritchie, 2019). Besides the high prevalence of micronutrients deficiencies, an analysis of food supply data (Michaelsen et al., 2011) suggests that the essential fatty acid intakes of pregnant and lactating women in some settings in SSA may be low.

It is likely that micronutrient deficiencies may contribute to some of the adverse health and nutrition outcomes among women and children in SSA. In 2015, an estimated 14% of infants born in the region had low birth weight (Blencowe et al., 2019) mostly due to fetal growth restriction linked to micronutrient deficiencies (UNICEF/WHO, 2019). Micronutrient deficiencies may also contribute to the relatively high rates of pregnancy complications (Darnton‐Hill & Mkparu, 2015), maternal mortality (Bailey, West, & Black, 2015), and impaired child growth and development in SSA.

Much of the micronutrient deficiencies among women and children in SSA may be attributed to low dietary intakes, likely as a result of over‐reliance on poor‐quality staple diets low in nutrient density (Bose, Baldi, Kiess, & de Pee, 2019; Deptford et al., 2018). For infants and young children, additional challenges exist, including relatively small gastric capacity, which limits how much can be consumed at a meal, and low meal frequency (Dewey, 2016). Meanwhile, nutrition‐specific interventions such dietary diversification (eg. promoting the consumption of locally available nutrient‐dense food groups), nutrient supplementation, bio‐fortification, and commercial fortification of staple foods may not always yield the desired results in SSA due to a variety of reasons including low affordability, particularly in deprived communities (Lalani, Bechoff, & Bennett, 2019), and low coverage rates of fortified foods (Univ Ghana/GroundWork/Univ Wisconsin‐Madison/KEMRI/UNICEF, 2017). In addition, it is difficult to deliver adequate amounts of iron and zinc in complementary foods for infants without any fortification, even when using a combination of several locally‐available high quality foods (Bose et al., 2019; Osendarp et al., 2016).

Reports from several studies in SSA (Adu‐Afarwuah et al., 2007; Adu‐Afarwuah et al., 2015; Adu‐Afarwuah et al., 2016; Hess et al., 2015) suggest that the use of small‐quantity lipid‐based supplements (SQ‐LNSs) to enrich home‐prepared foods for women and children might hold promise as one of possible interventions for delivering a large number of essential micro‐ and macro‐nutrients, and thereby addressing nutrient deficiencies, in these vulnerable groups. Two recent Cochrane reviews examined the impact of SQ‐LNS consumption among women (Das et al., 2018) and infants and young children 6‐23 mo of age (Das et al., 2019), but those reviews extended beyond SSA, and the possible reasons for the differential impact of SQ‐LNS consumption across different settings were rarely discussed. This paper aimed to examine the impact of SQ‐LNSs supplementation among women and children in SSA, and to discuss the possible factors related to the positive impact of SQ‐LNS supplementation in some, but not all, study contexts.

## 2 METHODS

In a narrative review, papers reporting randomized trials of SQ‐LNSs consumption among women and/or children in SSA were identified by electronically searching PubMed. Papers were selected if they contained “lipid‐based nutrient supplements” in the title, abstract or keywords list, and anthropometric, biochemical or child developmental outcomes were measured. The references in identified publications were also searched manually for papers that may not have appeared in the electronic search. Only studies conducted in SSA, in which the diets of apparently healthy women and/or children ≥ 6 mo of age were supplemented with SQ‐LNSs were reviewed.

This present paper examined the impact of SQ‐LNS supplementation on maternal anthropometric and hematologic indices, and on infant and child growth and development, for which considerable data have so far been published. Because the paper was based on a narrative review (rather than a systematic review), it is possible that certain relevant papers published at the time may not have been identified.

## 3 RESULTS

### 3.1 Small‐quantity lipid‐based nutrient supplements

Small‐quantity lipid‐based nutrient supplements (SQ‐LNSs) include the family of multiple micronutrient (MMN)‐fortified semi‐solid pastes typically given at a dose of about 20 g/d or 110 kcal/day for the purpose of preventing undernutrition (FANTA, 2016). Typically, they are prepared using vegetable oil, groundnut paste, milk, sugar, and specific amounts of micronutrients depending on the type of product and the target population, and they provide micronutrients as well as good quality protein and essential fatty acids, while leaving enough room for other foods in the diet (Arimond et al., 2015). When mixed with home‐prepared foods for women (e.g, during pregnancy and/or lactation) and children, SQ‐LNSs increase the nutrient intakes of these vulnerable groups and permit targeting to the individuals consuming them. Because of their low water activity (aw <0.5), SQ‐LNSs are not good substrates for the growth of bacteria, yeast and fungi (Arimond et al., 2015), and therefore do not require refrigeration for storage. SQ‐LNSs and multiple micronutrient powders (MNPs) or capsules can be similar in terms of micronutrient contents (De‐Regil, Suchdev, Vist, Walleser, & Pena‐Rosas, 2013; Suchdev, Pena‐Rosas, & De‐Regil, 2015), but they differ in the sense that SQ‐LNSs come from a food base and often contain energy, protein and essential fatty acids (EFA), as well as being able to include certain minerals (e.g. calcium, magnesium, phosphorus, and potassium) that cannot be incorporated in substantial amounts in MNPs. Other advantages of SQ‐LNSs are that they may enhance the taste and acceptability of cereal porridges commonly consumed in developing countries, and they may not adversely affect infant and young child feeding practices (Arimond et al., 2016).

Formulations of Small‐quantity lipid‐based nutrient supplements tested in SSA

SQ‐LNSs for infants 6‐18 mo of age and for pregnant and lactating women have so far been developed and used in several countries in SSA. In the case of SQ‐LNSs for infants, the various formulations include the Nutributter used in Ghana (Adu‐Afarwuah et al., 2007); those used in the iLNS trials in Ghana (Adu‐Afarwuah et al., 2016), Malawi (Ashorn et al., 2015a; Maleta et al., 2015), and Burkina Faso (Hess et al., 2015); and, more recently, those used in Kenya (Null et al., 2018) Zimbabwe (Humphrey et al., 2019) and South Africa (Smuts et al., 2019). These formulations usually contain up to about 22 vitamins and minerals, and are generally similar in terms of the daily dose being 20 to 25 g and providing 108‐130 kcal/d energy, as well as generally supplying 1*x* the WHO/FAO Recommended Nutrient Intake (RNI) for key micronutrients for infants 7–12 mo of age, and having as much of calcium, magnesium, phosphorus, and potassium as could be included given technical and organoleptic constraints. The main differences among the formulations usually relate to the iron and zinc contents, such as the 6 mg/d iron used in the iLNS DYAD and iLiNS ZINC trials (Adu‐Afarwuah et al., 2016; Ashorn et al., 2015a; Hess et al., 2015) compared with 9 mg/d iron used previously in Ghana (Adu‐Afarwuah et al., 2007) and more recently in Kenya (Null et al., 2018). In the South Africa study (Smuts et al., 2019) both SQ‐LNS and SQ‐LNS‐Plus (which contained soy and dairy protein but not groundnut paste) provided 50% of the Recommended Nutrient Intakes (RNIs) for children aged 1‐3 y of age for most of the micronutrients, and the latter included additional constituents namely choline, docosahexaenoic acid, arachidonic acid, L‐lysine, and phytase.

The SQ‐LNSs for pregnant and lactating women used in the iLiNS DYAD trials in Ghana (Adu‐Afarwuah et al., 2015) and Malawi (Ashorn et al., 2015b) were modeled on the International Multiple Micronutrient Preparation (UNIMMAP) (UNICEF/WHO/UNU, 1999) and a similar product used in Guinea Bissau (Kaestel, Michaelsen, Aaby, & Friis, 2005). This formulation provided approximately one Recommended Dietary Allowance (RDA) of 4 vitamins (A, D, folic acid, pantothenic acid) and one mineral (Manganese), and 2 RDAs of 7 vitamins (C, B1, B2, B3, B6, B12, E) and 3 minerals (zinc, copper, selenium), while vitamin K and iodine were based on current WHO recommendations, and iron was kept at 20 mg/day, a level the iLiNS Project researchers considered adequate for both pregnancy and lactation.

Randomized controlled trials of lipid‐based nutrient supplements in Sub‐Saharan Africa

Since the mid‐2000s, several randomized controlled trials in Sub‐Saharan Africa have evaluated the impact of SQ‐LNSs supplementation on key child and maternal outcomes (**Table** 1). In most of these studies, the SQ‐LNSs were provided post‐natally to children at or after 6 mo of age. Among these trials was an earlier trial in Ghana (Adu‐Afarwuah et al., 2007), which randomized 6‐mo‐old infants to consume either to a micronutrient powder, or a crushable micronutrient tablet, or SQ‐LNS daily until 12 mo old. In the Lungwena trial in Malawi (Phuka et al., 2008) infants were randomly assigned to consume either a fortified maize‐soy blend (71 g/d), or a medium‐quantity (50 g/d) lipid‐based nutrient supplement or SQ‐LNS (25 g/d) daily from 6 mo to 18 mo of age. The iLiNS‐DOSE trial in Malawi (Maleta et al., 2015) included 6‐mo‐old infants who were randomized into 1 of 6 groups to receive either 10 g/d, 20 g/d, or 40 g/d lipid‐based nutrient supplements (LNSs) containing milk powder; 20 g/d or 40 g/d LNS containing no milk‐powder; or no supplement until 18 mo of age. In the iLiNS‐ZINC trial in Burkina Faso (Hess et al., 2015), infants were randomized into a non‐intervention group, or 1 of 4 intervention groups in which they received SQ‐LNSs without zinc + placebo tablet, or SQ‐LNSs containing 5 mg zinc + placebo tablet, or SQ‐LNSs containing 10 mg zinc + placebo tablet, or SQ‐LNSs without zinc and 5 mg zinc tablet daily from 9 to 18 mo of age. For this Burkina Faso trial (Hess et al., 2015), children in the intervention groups, but not the non‐intervention group, received morbidity surveillance and treatment for diarrhea and malaria weekly.

**TABLE 1:** A summary of the methodology of supplementation trials in Sub‐Saharan Africa involving small‐quantity lipid‐based nutrient supplements (SQ‐LNSs) for the prevention of malnutrition^1^

Study location or name, Country (Period)	Study design	Study arms	Target group/age or status at start of intervention	Duration of intervention	Total number enrolled
1. Koforidua, Ghana (2004–2005)	RCT, researcher blinded to outcome	SQ‐LNS Micronutrient powder Crushable micronutrient tablet Control: No intervention	Children/ 6 mo	6 mo	409
2. Lungwena, Malawi (2004–2005)	RCT, researcher blinded to outcome	Medium‐quantity lipid‐based nutrient supplements SQ‐LNS Fortified corn‐soy blend	Children/ 6 mo	12 mo	182
3. iLiNS‐DOSE, Malawi (2009–2012)	RCT, researcher blinded to outcome	40 g SQ‐LNS not containing milk 40 g SQ‐LNS containing milk 20 g SQ‐LNS not containing milk 20 g SQ‐LNS containing milk 10 g milk SQ‐LNS containing milk Control: Delayed intervention, corn‐soy blend from 18 mo of age	Children/ 6 mo	12 mo	1,932
4. iLiNS‐ZINC, Burkina Faso (2010–2014)	Cluster‐ RCT, researcher partially blinded to outcome	SQ‐LNS containing no zinc SQ‐LNS containing 5 mg zinc SQ‐LNS containing 10 mg zinc SQ‐LNS containing no zinc + 5 mg zinc tablet Control: No intervention *All arms except the control arm received weekly morbidity surveillance and treatment for diarrhea and malaria	Children/ 9 mo	9 mo	3,220
5. WASH Benefits, Kenya (2012‐2014)	Cluster‐ RCT, researcher blinded to outcome	Drinking chlorinated water (Water group) Safe sanitation (Sanitation) Handwashing with soap (Handwashing) Combined water, sanitation, and handwashing (WASH) Complementary feeding counselling + SQ‐LNS to offspring from 6–24 mo (Nutrition) Combined water, sanitation, handwashing and nutrition (WASH + Nutrition) Active control: No intervention; monthly household visits to measure mid‐upper‐arm circumference (Active) Passive control: No intervention; visited for outcome data collection only at the same time as in other groups (Passive) *All intervention arms were visited monthly to measure mid‐upper‐arm circumference.	Women/2^nd^ or 3^rd^ trimester Children/6 mo	Varied in pregnancy through 24 mo postpartum 24 mo for children	8246 women, 7780 children
6. iLiNS‐DYAD‐G, Ghana (2009–2014)	RCT, partially double‐blind, researcher blinded to outcome	SQ‐LNSs to women until 6 mo postpartum; SQ‐LNSs to offpring from 6 to 18 mo of age (LNS group) Multiple micronutrient capsule to women until 6 mo postpartum; no supplementation to offspring from 6 to 18 mo of age (MMN) Iron‐folic acid to pregnant women and placebo during 6 mo postpartum; no supplementation to offspring from 6 to 18 mo of age (IFA)	Women/as early as possible in pregnancy Children/6 mo	Varied in pregnancy through 6 mo postpartum 12 mo for children	1,320 women, 1,185 children
7.iLiNS‐DYAD‐M, Malawi (2011–2015)	RCT, researcher blinded to outcome	SQ‐LNSs to women until 6 mo postpartum; SQ‐LNSs to offpring from 6 to 18 mo of age (LNS group) Multiple micronutrient capsule to women until 6 mo postpartum; no supplementation to offspring from 6 to 18 mo of age (MMN) Iron‐folic acid to pregnant women and placebo during 6 mo postpartum; no supplementation to offspring from 6 to 18 mo of age (IFA)	Women/as early as possible in pregnancy Children/6 mo	Varied in pregnancy, through 6 mo postpartum 12 mo for children	1,391 women, 869 children
8. WASH Benefits, Zimbabwe (2012‐2015)	Cluster‐ RCT, researcher blinded to outcome	Standard of care (promotion of exclusive breastfeed and uptake of government‐provided child‐care services). Complementary feeding counselling + SQ‐LNS to offspring from age 6 to 18 mo of age (IYCF) Water, sanitation and hygiene (WASH) IYCF plus WASH	Women/2^nd^ or 3^rd^ trimester Children/6 mo	Varied in pregnancy through 18 mo post‐partum 18 mo for children	5280 women, 3989 children
9. Jouberton, South Africa (2013‐2015)	RCT, researcher partially blinded to outcome	SQ‐LNS SQ‐LNS‐plus (included added choline, arachidonic acid, docosahexaenoic acid, L‐lysine, and phytase) to children No supplementation (i.e. SQ‐LNSs after completion of the trial)	Children/6 mo	6 mo	750

1 Small‐quantity lipid‐based nutrient supplements (SQ‐LNSs) generally given at a daily dose of 20 – 25 g and providing 108‐130 kcal/d energy.

The so‐called WASH Benefits cluster‐randomized trial in Kenya (Null et al., 2018) involved 8 groups, including an active control group, a passive control group, and 6 intervention groups, namely: water; sanitation; handwashing; combined water, sanitation, and handwashing; nutrition; and combined water, sanitation, handwashing, and nutrition. In the 2 intervention groups that included nutrition, children born to the women received SQ‐LNS from 6 to 24 mo of age as part of the intervention package. In a similar WASH Benefits cluster‐randomized trial in Zimbabwe (Humphrey et al., 2019), pregnant women were assigned to 1 of 4 groups including: standard of care; infant and young child feeding (IYCF); provision of water, sanitation and hygiene (WASH); and combined IYCF plus WASH. In the 2 group that included IYCF, children born to the participating women received SQ‐LNSs from 6 to 18 mo of age. Data analysis included a comparison of the 2 group that included IYCF groups versus the 2 non‐IYCF groups. In South Africa (Smuts et al., 2019), infants were randomized to receive daily, a more regular type of SQ‐LNS or SQ‐LNS‐Plus or no supplementation from 6 to 12 mo of age.

There are 2 studies thus far in Sub‐Saharan Africa in which SQ‐LNSs were provided to women during pregnancy and lactation, and to the offspring during complementary feeding (i.e much of the “first 1000 days”). In the iLiNS‐DYAD trials in Ghana (Adu‐Afarwuah et al., 2015; Adu‐Afarwuah et al., 2016) and Malawi (Ashorn et al., 2015a; Ashorn et al., 2015b), women ≤ 20 weeks pregnant were randomized to receive either 60 mg/d iron + 400 μg/d folic acid (IFA group), 1 capsule/d containing 18 micronutrients (MMN group), or 20 g/d SQ‐LNS with 22 micronutrients (LNS group) during pregnancy, followed by placebo (200 mg Ca/d), MMN or SQ‐LNS, respectively, during the first 6 mo postpartum. The infants born to women in the LNS group received SQ‐LNS designed for infants from 6 to 18 mo of age, but their counterparts in the other 2 groups received no supplementation.

### 3.2 Impact of SQ‐LNS supplementation on maternal anthropometric and hematologic outcomes

- Impact on maternal anthropometric outcomes

The iLiNS DYAD study in Ghana (Adu‐Afarwuah, Lartey, Okronipa, Ashorn et al., 2017a) reported the impact of SQ‐LNS supplementation given during pregnancy and the first 6 mo postpartum on women's anthropometric outcomes. At 36 weeks’ gestation, women who received iron and folic acid (IFA group), or multiple micronutrients (MMN group), or SQ‐LNSs (LNS group) from early pregnancy (when mean gestational age was 16.1 weeks) did not differ significantly in most of the anthropometric outcomes measured. Using the Institute of Medicine's guidelines, the prevalence of inadequate gestational weight gain (GWG) based on estimated pre‐pregnancy values was significantly lower in the LNS group (57.4%) compared with the MMN (67.2%), but not the IFA (63.1%) groups. In analysis involving the LNS group versus IFA and MMN groups combined (non‐SQ‐LNSs), mean ± SD percent adequacy of GWG was significantly greater, the prevalence of inadequate GWG was significantly lower, and the prevalence of adequate GWG was significantly greater in the LNS group than the non‐LNS group. At 6 mo postpartum, groups did not differ in mean ± SD weight, mid‐upper arm circumference, triceps skinfold measurements, and body mass index or changes in these indices from estimated pre‐pregnancy values, nor in the percentage of women with excessive GWG, or the percentage of women who had normal BMI at pre‐pregnancy but who became overweight or obese by 6 mo postpartum (Adu‐Afarwuah, Lartey, Okronipa, Ashorn et al., 2017a).

- Impact on maternal hematologic outcomes

The hematologic indices reported from the iLiNS‐DYAD Ghana trial (Adu‐Afarwuah, Lartey, Ashorn, Zeilani et al., 2017b) showed that compared with the women assigned to iron and folic acid (IFA group), those assigned to the SQ‐LNSs or multiple micronutrients had significantly lower mean hemoglobin concentration (120 ± 11 vs. 115 ± 12 and 117 ± 12, respectively; P < 0.001), higher mean zinc protoporphyrin, ZPP (42 ± 30 vs. 50 ± 29 and 49 ± 30; P = 0.010) and transferrin receptor, TfR (4.0 ± 1.3 vs. 4.9 ± 1.8 and 4.6 ± 1.7; P < 0.001), and greater prevalence of anemia (2.2% vs. 7.9% and 5.8%; P = 0.019), elevated ZPP (>60) [9.4% vs. 18.6% and 19.2%; P = 0.003] and elevated TfR (>6.0) [9.0% vs. 19.2% and 15.1%; P = 0.004] at 36 weeks of gestation.

Impact of SQ‐LNS supplementation on fetal growth and children's post‐natal growth and developmental outcomes

- Impact on fetal growth

SQ‐LNS supplementation during pregnancy had an impact on fetal growth in Ghana (Adu‐Afarwuah et al., 2015) but not in Malawi (Ashorn et al., 2015b). In the Ghana study (Adu‐Afarwuah et al., 2015), pre‐natal SQ‐LNS, compared with iron and folic acid, supplementation significantly increased infants’ mean birth weight by 85 g, mean weight‐for‐age z‐score by 0.19 and mean BMI‐for‐age z‐score by 0.21; and reduced the relative risk (RR) of being born low birthweight by 39% (RR: 0.61, 95% CI: 0.39, 0.96; P = 0.032. Among first‐time mothers, the consumption of SQ‐LNS during pregnancy increased birth length and birth weight by 0.91 cm and 237 g, respectively, and reduced the RRs of low birthweight (LBW) by 69% and of small‐for‐gestational age (SGA) by 44%, when compared with iron and folic supplementation. Compared with multiple micronutrients supplementation, these differences were 0.67 cm, 139 g, and the reductions were 53% in LBW and 34% in SGA. No significant group differences were found in multiparous women.

- Impact on post‐natal growth

All the trials in which SQ‐LNS supplementation was provided to children ≥ 6 mo of age in Sub‐Saharan Africa evaluated children's growth during the period 11‐24 mo of age (Das et al., 2019; FANTA, 2016). In 3 studies in Malawi, including the Lungwena (Phuka et al., 2008), the iLiNS‐DOSE (Maleta et al., 2015) and the iLiNS‐DYAD (Ashorn et al., 2015a) trials, SQ‐LNSs supplementation had no impact on fetal or child growth, except for the Lunguena trial (Phuka et al., 2008), in which children consuming SQ‐LNS (as well as those consuming medium‐quantity lipid‐based nutrient supplement with a dose of 50 g/d) from 6 to 18 mo of age had significantly lower cumulative 12‐month incidence of severe stunting (3.5% and 0%, respectively) compared to those who consumed the maize‐soy blend (13.3%).

In contrast to the results from Malawi (Ashorn et al., 2015a; Maleta et al., 2015), the provision of SQ‐LNSs to infants had a significant impact on mean anthropometric outcomes in several settings in SSA. In Ghana (Adu‐Afarwuah et al., 2007), the infants who consumed SQ‐LNSs from 6 to 12 mo of age had greater mean ± SD length‐for‐age, LAZ (‐0.20 ± 0.54) and weight‐for‐age, WAZ (‐0.49 ± 0.54) z‐scores, compared with infants assigned to the crushable micronutrient tablets group (LAZ: ‐0.39 ± 0.54; WAZ:‐0.67 ± 0.54) or to the crushable micronutrient tablets and micronutrient power groups combined (LAZ: ‐0.38 ± 0.54; WAZ: ‐0.65 ± 0.54).

Similarly, in the more recent iLiNS DYAD Ghana trial (Adu‐Afarwuah et al., 2016), children who received SQ‐LNSs from 6 to 18 mo of age as did their mothers from pregnancy to 6 mo postpartum had greater attained mean ± SD length (79.7 ± 2.9 cm) and weight (9.9 ± 1.2 kg) and greater mean ± SD of the corresponding LAZ and WAZ values by 18 mo of age than children in the other two groups combined (79.1 ± 2.9 cm and 9.7 ± 1.2 kg, respectively), who received no supplementation and their mothers were assigned to iron and folic acid during pregnancy only, or to multiple micronutrients during pregnancy and the first 6 mo postpartum.

In the iLiNS‐ZINC trial in Burkina Faso (Hess et al., 2015), infants who received SQ‐LNSs with varied amounts of zinc, along with morbidity surveillance and treatment for malaria and diarrhea from 9 to 18 mo of age had greater mean ± SD length (77.7 ± 3.0 vs. 76.9 ± 3.4 cm; p<0.001) and weight (9.3 ± 1.1 vs 9.0 ± 1.2 kg; p < 0.001) and a lower prevalence of stunting (29.3% vs 39.3%; p < 0.001) than their counterparts who receive no SQ‐LNSs, tablets, illness surveillance or treatment.

Results from the WASH Benefits trial in Kenya (Null et al., 2018) showed that compared with children in the active control group who had a mean LAZ of –1.54, those in the SQ‐LNS (Nutrition) and those in the WASH + Nutrition groups were taller by year 2 of follow up, with difference in mean LAZ (95% CI) being 0.13 (0.01, 0.25) and 0.16 (0.05, 0.27), respectively. None of the intervention treatments which did not include the provision of SQ‐LNSs had any effect on linear growth. These results paralleled those from Zimbabwe (Humphrey et al., 2019), where mean LAZ (95% CI) was 0.16 (0.08, 0.23) greater, and the relative risk of stunting was 21% lower at 18 mo of age for children in the IYCF groups (in which SQ‐LNSs were provided), compared with children in the non‐IYCF groups who consumed no SQ‐LNS. Similarly, in South Africa (Smuts et al., 2019), the provision of SQ‐LNS with added choline, docosahexaenoic acid, arachidonic acid, lysine, and phytase (ie. SQ‐LNS‐Plus) from 6 to 12 mo, compared with no supplementation, increased LAZ (95% CI) at 8 mo of age by 0.11 (0.01, 0.22) and LAZ (95% CI) at 10 mo of age by 0.16 (0.04, 0.27), although the difference in LAZ at 12 mo of age compared to the control (0.09; 95% CI: ‐0.02, 0.21), was not statistically significant. Investigators speculated that the other SQ‐LNS product with no added choline, docosahexaenoic acid, arachidonic acid, lysine, and phytase produced no impact on linear growth possibly because without such added constituents, the 50% of RNIs for children 1‐3 y of age for most of the micronutrients provided in that formulation were insufficient to sustain substantial growth.

- Infant development

Several SQ‐LNS supplementation trials carried out in SSA have reported various infant developmental outcomes. In the Lungwena trial in Malawi (Phuka et al., 2012) the consumption of SQ‐LNS from 6 to 18 mo of age had no impact on any of the Griffiths’ developmental scores, including mean ± SD raw developmental scores, developmental quotients, and mental age, as well as any of the developmental subscales measured. Likewise, the iLiNS‐DYAD trials in Ghana (Prado, Adu‐Afarwuah, Lartey, Ocansey, Ashorn, Vosti, & Dewey, 2016) and Malawi (Prado, Maleta, Ashorn, Ashorn, Vosti, Sadalaki, & Dewey, 2016) reported no significant group differences in language, socio‐emotional, or executive function at 18 mo of age. In contrast to these reports (Prado, Adu‐Afarwuah et al., 2016, Phuka et al., 2012, Prado, Maleta et al., 2016), the provision of SQ‐LNSs to infants from age 9 to 18 mo, along with malaria and diarrhea treatment in Burkina Faso (Prado, Abbeddou, Yakes Jimenez, Some, Ouedraogo, Vosti, Dewey, Brown, Hess, & Ouédraogo, 2016) resulted in greater mean scores language, and personal‐social development.

The impact of SQ‐LNS consumption on the achievement of motor milestones was evaluated in Ghana (Adu‐Afarwuah et al., 2007, Prado, Adu‐Afarwuah et al., 2016), Burkina Faso (Prado, Abbeddou et al., 2016) and Malawi (Prado, Maleta et al., 2016). In Ghana, SQ‐LNS supplementation (given to infants alone or to both mothers and infants) was associated with a greater percentage of infants being able to walk independently by 12 mo of age in an earlier study (Adu‐Afarwuah et al., 2007) when compared with children who received no intervention, as well as in the iLiNS‐DYAD trial (Prado, Adu‐Afarwuah et al., 2016) when compared with children in the IFA group. Similarly, Burkinabe children who received SQ‐LNS supplementation together with morbidity surveillance and treatment for diarrhea and malaria (Prado, Abbeddou et al., 2016) had significantly greater mean motor development scores (indicating higher performance) than children who received no intervention. In Malawi, the supplementation of infants’ diet with SQ‐LNSs had no impact on locomotor score in the Lungwena study (Phuka et al., 2012), but in the iLiNS‐DYAD trial (Prado, Maleta et al., 2016), children in the LNS group reportedly achieved standing with assistance earlier than those in the MMN group (B = 0.51; 95% CI: 0.12, 0.89; P = 0.029), and achieved waving goodbye (B = 0.60; 95% CI: 0.12, 1.08; P = 0.040) and walking alone (B = 0.53; 95% CI: 0.11, 0.94; P = 0.034) earlier than those in the IFA group. Furthermore, on the basis of researcher observation, there was a trend (P = 0.052) towards a greater percentage of children in the LNS group (58%) being able to walk independently by 12 mo age compared with children in the IFA (49%) and MMN (49%) groups.

In the WASH Benefits study in Kenya (Stewart et al., 2018), the supplementation of infants’ diet with SQ‐LNSs may have contributed to a significant positive impact on children's gross motor milestone achievement by year 1, although it did not affect gross motor (or any of the developmental outcomes measured) by year 2. Specifically, children in the WASH + Nutrition group had greater rates of achieving the standing with assistance milestone (hazard ratio 1.23; 95% CI: 1.09, 1.40) and the walking with assistance milestone (1.32; 95% CI: 1.17, 1.50) by year 1, when compared with children in the active control group. Mixed results were found in South Africa (Smuts et al., 2019): when compared with children in the control group who received no supplementation, those in the SQ‐LNS‐Plus group did not differ in the age of achieving the walking alone milestone, but had greater locomotor development score (2.05; 95% CI: 0.72, 3.38; P = 0.003) as well as Parent Rating Score (gross motor development) (1.10; 95% CI: 0.14, 2.07; P = 0.025).

## 4 DISCUSSION

This paper examines the results from randomized trials conducted in SSA, in which the diets of apparently healthy women and/or children ≥6 mo of age were supplemented with SQ‐LNSs. In Ghana, prenatal SQ‐LNS consumption reduced the prevalence of low gestational weight gain (GWG) when compared with multiple micronutrients supplementation (Adu‐Afarwuah, Lartey, Okronipa, Ashorn et al., 2017a), and was associated with poorer iron and hemoglobin status when compared with iron and folic acid supplementation (Adu‐Afarwuah, Lartey, Ashorn, Zeilani et al., 2017b). The provision of SQ‐LNSs given alone (Adu‐Afarwuah et al., 2007; Adu‐Afarwuah et al., 2015; Adu‐Afarwuah et al., 2016; Ashorn et al., 2015a; Ashorn et al., 2015b; Maleta et al., 2015; Smuts et al., 2019) or as part of a package of interventions (Hess et al., 2015; Humphrey et al., 2019; Null et al., 2018) was associated with improved infant or child growth in two trials in Ghana (Adu‐Afarwuah et al., 2007; Adu‐Afarwuah et al., 2015; Adu‐Afarwuah et al., 2016), and one trial each in Burkina Faso (Hess et al., 2015), Kenya (Null et al., 2018), Zimbabwe (Humphrey et al., 2019) and South Africa (Smuts et al., 2019), but had no impact on infant or child growth in two trials in Malawi (Ashorn et al., 2015a, Ashorn et al., 2015b, Maleta et al., 2015). Likewise, the impact of SQ‐LNSs supplementation on various child development outcomes have been mixed: no effect was found on Griffiths’ developmental scores in Malawi (Phuka et al., 2012), or on language, socio‐emotional, and executive functions in Ghana (Prado, Adu‐Afarwuah et al., 2016) and Malawi (Prado, Maleta et al., 2016), but an association with improved motor development was found in Ghana (Adu‐Afarwuah et al., 2007, Prado, Adu‐Afarwuah et al., 2016), Burkina Faso (Prado, Abbeddou et al., 2016), Malawi (Prado, Maleta et al., 2016), Kenya (Stewart et al., 2018) and South Africa.

The results regarding pre‐natal SQ‐LNSs consumption and women's anthropometric and hematologic outcomes in Ghana (Adu‐Afarwuah, Lartey, Okronipa, Ashorn et al., 2017a; Adu‐Afarwuah, Lartey, Ashorn, Zeilani et al., 2017b) warrant a discussion. First, the small extra energy and the milk contents of the SQ‐LNSs may have contributed to the reduced prevalence of inadequate GWG in the LNS group, as evidenced by other investigations (Institute of Medicine and National Research Council, 2007; Ramakrishnan, 2004) showing that a higher energy intake and the consumption of dairy during pregnancy may be associated with a lower risk of inadequate gestational weight gain (as well as a higher risk of excessive gestational gain). Second, it is possible that the 20 mg/d iron dose in the SQ‐LNS formulation may have been too low for the population (Mei et al., 2014; Roberfroid et al., 2011), or that, the relatively high dose of zinc (30 mg) in the formulation may have interfered with iron absorption, as suggested by results of a similar supplementation trial in Nepal (Christian et al., 2003).

The positive impact of prenatal SQ‐LNSs consumption on mean birthweight of offspring in the Ghanaian sample supports the notion that in that population, the provision of macronutrients (e.g. EFAs) along with multiple micronutrients was necessary to increase fetal growth. As it was shown previously (Michaelsen et al., 2011), the estimated n‐3 fatty acid supply in the diet of Ghanaian pregnant women may be low. The stronger impact of SQ‐LNSs consumption among primiparous (or younger) women in Ghana suggests that this intervention my offset some of the vulnerabilities associated with primiparity when compared with multiparity (Kramer, 1987). In Ghana, these vulnerabilities appeared to include lower mean hemoglobin concentration, and greater likelihood to be anemic or test positive for malaria at the study enrollment.

Regarding the conflicting results from the Malawi trials (Ashorn et al., 2015a; Ashorn et al., 2015b; Maleta et al., 2015) compared with those from other settings in Ghana (Adu‐Afarwuah et al., 2007; Adu‐Afarwuah et al., 2015; Adu‐Afarwuah et al., 2016), Burkina Faso (Hess et al., 2015), Kenya (Null et al., 2018), Zimbabwe (Humphrey et al., 2019) and South Africa (Smuts et al., 2019) with respect to infant and child growth, these findings suggest that the success of SQ‐LNS supplementation in promoting fetal and child growth in SSA may be context‐specific (Manary, 2015). It appears that the SQ‐LNS is more likely to have an impact in settings where constraints on child growth eg. short maternal stature, poor access to health care, high rates of infections, etc. are fewer. In Malawi, children's linear growth may have been restricted by high rates of asymptomatic infections, environmental enteropathy, HIV infection and short maternal stature. (Ashorn et al., 2015b). Comparatively, in a peri‐urban setting in Peru (Penny et al., 2005) with a relatively low prevalence of stunting at 18 mo, a nutrition education intervention had a positive impact on stunting, compared to a control group.

## 5 CONCLUSIONS

The provision of SQ‐LNSs to pregnant women could be one potential strategy to address inadequate gestational weight gain (GWG) in SSA, without increasing the risk of excessive GWG, or overweight or obesity during the first 6 mo postpartum. Consequently, SQ‐LNSs may be added to the list of interventions for improving fetal growth in vulnerable populations in SSA. Further research on the amount of iron in SQ‐LNSs that is most effective for improving both maternal hemoglobin/iron status and birth outcomes is warranted. Regarding the impact of SQ‐LNS supplementation on child growth in SSA, results have been generally positive, though modest. SQ‐LNSs interventions might have a greater impact if they address the complex array of underlying factors that contribute to growth faltering.

## CONFLICTS OF INTEREST

The authors declare that they have no conflicts of interest.

## CONTRIBUTIONS

SA‐A wrote the manuscript and approved its final version.
